# Prevalence of post-COVID-19 in patients with fibromyalgia: a comparative study with other inflammatory and autoimmune rheumatic diseases

**DOI:** 10.1186/s12891-022-05436-0

**Published:** 2022-05-19

**Authors:** Javier Rivera, Tamara Rodríguez, Marta Pallarés, Isabel Castrejón, Teresa González, Laura Vallejo-Slocker, Juan Molina-Collada, Fernando Montero, Anna Arias, Miguel A. Vallejo, Jose M. Alvaro-Gracia, Antonio Collado

**Affiliations:** 1 Rheumatology Department, Hospital General Universitario Gregorio Marañón. Instituto de Investigación Sanitaria Gregorio Marañón (IiSGM), Francisco Silvela 40, 28028 Madrid, Spain; 2 GEFISER, Sociedad Española de Reumatología, Barcelona, Spain; 3 Rheumatology Department, Hospital Clínic de Barcelona, Madrid, Spain; 4 Department of Clinical Psychology, National Distance Education University (UNED), Madrid, Spain

**Keywords:** Fibromyalgia, COVID-19, Post COVID-19, Long COVID-19, Rheumatic diseases

## Abstract

**Objectives:**

To determine the prevalence and characteristics of post-COVID-19 (PC) in fibromyalgia (FM) patients.

**Methods:**

Retrospective, multi-centric, observational study, comparing a group of FM patients (FM group) with another group of patients with other rheumatic diseases (RD group). COVID-19 diagnosis was established by positive polymerase chain reaction or antigen during acute infection or by positive antibodies thereafter. We considered PC diagnosis when symptoms remain after COVID-19. We collected the principal characteristics of COVID-19, the severity of fatigue, waking unrefreshed and cognitive impairment, and persistent symptoms. The American College of Rheumatology (ACR) criteria and the Combined Index of Severity in Fibromyalgia (ICAF) were collected in the FM group.

**Results:**

RD group (*n* = 56) had more pneumonia (*p* = 0.001) and hospital admissions (*p* = 0.002), but the FM group (*n* = 78) had a higher number of symptoms (*p* = 0.002). The percentage of patients with PC was similar between groups (FM group 79.5%; RD group 66.1%, *p* = 0.081). FM group had more PC symptoms (*p* = 0.001), more impairment after COVID-19 (*p* = 0.002) and higher severity of fatigue, waking unrefreshed and cognitive impairment (*p* <  0.0001). Only loss of smell was more frequent in the FM group (*p* = 0.005). The FM group with PC (*n* = 29) showed more severity of the Combined Index of Severity in Fibromyalgia (ICAF) total score and physical factor after COVID-19, while emotional, coping factors and the ACR criteria did not change.

**Conclusions:**

The prevalence of PC in FM patients is similar to RD patients. In FM patients, the presence of PC does not appear to impact the severity of FM.

## Background

The clinical picture known as post-COVID-19 (PC) or long-COVID-19 [1] has been described in the literature as the persistence of the symptoms long after the acute phase of the severe acute respiratory syndrome coronavirus 2 (SARS-CoV-2) infection [2]. The prevalence of PC is not well known because it depends on different criteria or the time frame of follow-up when the analysis is performed. In a recent review it is estimated that between 40 and 80% of the patients still remain symptomatic 2 months after COVID-19 [3, 4]. Patients with PC have a wide range of symptoms including extreme fatigue, cognitive alterations, musculoskeletal pain, breathlessness, headaches, sleep disorders, anxiety and depression, and loss of smell and taste [5, 6]. The total number of symptoms reported by patients may be much higher [7]. The clinical picture associated with a poor quality of life and a severe impairment of the functional capacity in patients with PC may resemble fibromyalgia (FM) or myalgic encephalomyelitis/chronic fatigue syndrome (ME/CFS), and this led us to hypothesize whether PC might be related to these diseases. The infectious theory in the aetiopathogenesis of ME/SFC and FM has been postulated and several viruses and different bacterial infections have been investigated, with no concluding evidence so far [8, 9]. Interestingly, the 2003 SARS-COV-1 infection in East Asia, produced by another coronavirus, triggered in some of the patients a post infectious syndrome with chronic widespread musculoskeletal pain, fatigue, depression and sleep disorders similar to FM and ME/CFS [10]. A long lasting similar clinical picture has also been observed in other viral and non-viral infections [11], suggesting a common pathogenetic pathways in these diseases.

As PC and FM may share several symptoms, our hypothesis is that patients with an established FM may have a higher probability to develop PC after an infection by SARS-COV-2. We compared the patients of FM group with a group of patients with inflammatory rheumatic diseases (RD), but not with healthy controls, because RD patients may develop FM more frequently than healthy people, probably due to the inflammatory disease itself.

The aim of this study is to investigate the prevalence and the clinical characteristics of PC in FM patients in comparison with other RD patients.

## Methods

### Study design

This is a retrospective, case control, observational study conducted in two tertiary care university teaching hospitals in Spain (Hospital Universitario Gregorio Marañón, Madrid; Hospital Clinic, Barcelona), including patients with FM compared with a group of patients with other inflammatory or autoimmune RD.

### Patients

Consecutive patients attending the outpatient clinics of each Rheumatology Department were invited to participate in the study if they had previously suffered COVID-19. After obtaining the patient consent form, we split the participants into two groups:

FM group. Patients with a diagnosis of FM who fulfilled the classification criteria of the American College of Rheumatology (ACR) 2010 [12].

RD group. Patients coming from the same outpatient clinics with a diagnosis of inflammatory or autoimmune diseases who fulfilled the diagnostic criteria for the corresponding disease.

Patients of both groups were included if they also met the following criteria: (a) female, (b) at least 18 years old, and (c) having been infected by the SARS-COV-2 any time since the beginning of pandemic.

The diagnosis of SARS-COV-2 infection was established by a positive polymerase chain reaction test on respiratory samples or a positive antigen test during the acute phase of the disease or by the presence of antibodies against SARS-COV-2 during the study frame in unvaccinated patients. Patients with a clinical picture suggesting COVID-19 but with PCR negative test, antigen negative test or absence of antibodies were excluded. PC diagnosis was established according to the following criteria: persistent symptoms of COVID-19 as well as symptoms which increased after the acute phase of the infection.

### Data collection

Data from a well-established clinical registry of patients with FM were complemented with additional data obtained by a personal interview. The main characteristics of the clinical picture during COVID-19 infection were obtained including symptoms reported by the patient, the presence of x-ray pneumonia and if the patient was admitted to hospital. At the time of the interview at the outpatient clinic we also registered the time (months) elapsed since the acute infection, the patient global impression of change (PGIC) [13] in relation to the period before COVID-19, the severity of three principal symptoms (fatigue, waking unrefreshed and cognitive impairment). We also registered the symptoms that persisted after COVID-19 infection. In the FM group, we also collected the widespread pain index (WPI) and the symptoms severity scale (SSS) of the ACR classification criteria [12], and FM patients completed the Combined Index of Severity in Fibromyalgia (ICAF) [14, 15].

In the HGUGH centre participating in the study, we established a cohort of FM patients who are annually followed including the ICAF questionnaire (ICAF cohort). In those patients with PC of this ICAF cohort, we compared the severity of FM by means of the ICAF questionnaire as well as the components of the ACR criteria before and after the onset of COVID-19.

### Questionnaires

ICAF [14, 15] is a specific 59-item quality-of-life questionnaire for FM patients. It offers a total score of the severity of the disease along with four other factors. The physical factor measures the functional ability from the point of view of physical capacity to perform daily activities; the emotional factor measures the severity of clinical manifestations such as anxiety and depression; and two factors of active and passive coping measure the attitude of the patient in coping with their disease. All scores are expressed on a T-scale with an average of 50 and a standard deviation of 10. A high score indicates greater severity, with the exception of the active coping factor that indicates a better coping. The importance of each factor over the total score is different, so the emotional and physical factors are the ones with the highest weight while each coping factor only contributes a small part of the total score severity of the disease [14]. ICAF can also assess the predominance of the type of coping used by patients based on higher scores in active or passive coping. With physical and emotional factors, the predominance of one over the other can also be assessed. This questionnaire has been previously validated in our FM population obtaining the following values: emotional factor: 50.7; physical factor: 54.3; active coping: 49.0; passive coping: 51.6, and total score: 52.5 [15].

The PGIC [13] is a specific questionnaire to evaluate overall improvement after an intervention. It is composed of a single Likert scale from 1 to 7 (1 = much better, 2 = better, 3 = a little better, 4 = equal, 5 = a little worse, 6 = worse or 7 = much worse) to assess how the patient is feeling after an intervention.

The ACR criteria [12, 16] are constructed with two variables: the widespread pain index (WPI) (range, 0–19), and the symptomatic severity scale (SSS) (range, 0–12). The sum of both constitutes the polysymptomatic distress scale (PSD) (range, 0–31) [16] which measures the global severity of FM.

The SSS itself is the sum the of 0–3 severity scores of the three principal symptoms: fatigue, waking unrefreshed, and cognitive symptoms (total score, 0–9), plus the sum of the presence of headaches, pain or cramps in lower abdomen and depression (total score, 0–3).

In the RD group, we only measured the severity score of the three principal symptoms (fatigue, waking unrefreshed and cognitive impairment) to establish the comparison with the FM group of patients.

### Statistical analysis

We used the t-test to compare quantitative variables and the chi-square test for nominal variables. We also used ANOVA for repeated measurements to analyze the evolution between the ICAF before and after COVID-19.

### Ethical approval

This study was performed in accordance with the ethical standards of the responsible committee on human experimentation and the Helsinki Declaration of 1975, as revised in 2013. All data were anonymized, and the study was approved by the Ethics Committee of Hospital General Universitario Gregorio Marañón (08/2021; April 6th, 2021), Instituto de Investigación Sanitaria Gregorio Marañón (IiSGM). Madrid. Spain.

## Results

A total of 78 FM and 56 RD female patients who had suffered COVID-19 since the beginning of pandemic and fulfilled the remaining inclusion criteria were included in the study.

The RD group included patients with different inflammatory and autoimmune diseases, as described in Table 1.

**Table 1:** Patients included in the RD group. Diagnosis, total number of the patients and percentage

Diagnosis	Number of patients	Percentage
Rheumatoid arthritis	26	46
Psoriatic arthropathy	9	17
Systemic lupus erythematosus	8	14
Ankylosing spondylitis	4	7
Systemic sclerosis	3	5
Primary Sjögren syndrome	3	5
Vasculitis	1	2
Undifferentiated connective disease	1	2
Primary antiphospholipid syndrome	1	2
Total	56	100

Mean age was 51.14 (9.78) in the FM group and 52.45 (11.01) in the RD group, without differences between groups. With respect to the severity of COVID-19, patients in the RD group had a more severe disease with a double case of pneumonia with x-ray confirmation (50% versus 23%, *p* = 0.001) and requiring hospital admission more frequently (37.5% versus 14.1%, *p* = 0.002) than the FM group patients (Table 2). However, the number of symptoms reported by the patients during the acute phase of the infection was significantly higher in the FM group (*p* = 0.002) (Table 2).

**Table 2:** Severity of COVID-19. Main characteristics of the acute phase of COVID-19 in both groups of patients

	FM Group	RD Group	*p*
x-ray pneumonia N^a^ (%)	18 (23.1)	28 (50.0)	0.001
Admission to the hospital N^a^ (%)	11 (14.1)	21 (37.5)	0.002
Number of symptoms mean (SD)^b^	5.33 (2.14)	4.18 (2.09)	0.002

^a^ *N* Number of patients. ^b^ *SD* Standard deviation

The mean time from the acute phase of the disease to the inclusion in the study was 8.50 (4.27) months in the FM group and 11.51 (3.41) months in the RD group (*p* <  0.0001). Although there was no statistical difference between groups (*p* = 0.081), the higher percentage of patients with PC in the FM group indicates a trend (Table 3). However, the patients of the FM group reported persistence of more symptoms (*p* = 0.001) and a poorer perception of PGIC after COVID-19 (*p* = 0.002). The severity of fatigue, waking unrefreshed and cognitive impairment of the ACR criteria was also significantly higher in comparison with patients of the RD group (*p* < 0.0001) (Table 3).

**Table 3:** Post-COVID-19 in both groups of patients. Main characteristics of the post-COVID-19 in both groups

	FM Group	RD Group	*p*
Prevalence of post-COVID-19 N^a^ (%)	62 (79.5)	37 (66.1)	0.081
Number of symptoms mean (SD)^b^	2.42 (1.84)	1.39 (1.39)	0.001
PGICb^c^ mean (SD)^b^	5.56 (1.35)	4.75 (1.57)	0.002
Severity of the three principal symptoms^d^ mean (SD)^b^	7.05 (1.58)	3.75 (2.54)	< 0.0001

^a^ *N* Number of patients. ^b^ *SD* Standard deviation. ^c^ *PGIC* Patient global impression of change. ^d^ Principal symptoms: fatigue, waking unrefreshed and cognitive impairment

A total of 99 (74%) patients from both groups presented PC. The most frequent symptoms of PC reported by the patients are described in Table 4. Although there were more symptoms in the FM group, only loss of smell reached a statistically significant difference: 25 (40.3%) in FM group versus 5 (13.5%) in RD group, *p* = 0.005.

**Table 4:** Symptoms of post-COVID-19. Most frequently reported symptoms of post-COVID-19 in all patients

	Number of patients	%
Fatigue	47	47.5
Musculoskeletal pain	36	36.4
Dyspnea	31	31.3
Loss of smell	30	30.3
Loss of taste	21	21.2
Headache	21	21.2
Cough	12	12.1
Cognitive impairment	10	10.1
Sleep disorders	7	7.1
Digestive disorders	6	6.1
Alopecia	6	6.1
Arthritis	4	4.0
Muscular spasms	4	4.0

In the analysis of the 29 FM patients from the ICAF cohort, patients with PC reported to be worse after the infection and the scores of ICAF showed higher values (more severity) for the total score and the physical factor, while the emotional factor and the coping factors did not change (Table 5). Also, the components of the ACR criteria did not show any statistical difference before and after the onset of COVID-19 (Table 5).

**Table 5:** FM patients with post-COVID-19. Evolution of the severity of FM before and after COVID-19 in those patients from the ICAF cohort with concomitant PC

	Before COVID-19		After COVID-19			ANOVA	
	Mean	(SD)^a^	Mean	(SD)^a^	F^b^	DF^c^	*p*
**ICAF**^d^							
Total	48.76	(10.31)	53.29	(9.39)	6.99	1,25	0.014
Physical factor	50.45	(10.83)	56.81	(8.30)	10.92	1,25	0.003
Emotional factor	48.39	(10.07)	51.14	(9.50)	3.94	1,25	0.058
Active coping	52.50	(9.92)	50.64	(11.49)	0.74	1,25	0.398
Passive coping	51.34	(10.55)	53.56	(12.69)	0.88	1,25	0.357
**ACR**^e^ **criteria**							
WPI^f^	12.90	(3.61)	13.76	(3.54)	2.08	1,28	0.160
SSS^g^	9.14	(2.73)	9.00	(2.39)	0.04	1,28	0.745
PSD^h^	22.03	(5.60)	22.76	(5.48)	0.73	1,28	0.398

^a^ *SD* Standard deviation. ^b^ *F* value. ^c^ *DF* Degree of freedom. ^d^ *ICAF* Combined Index of Severity of Fibromyalgia. ^e^ *ACR* American College of Rheumatology criteria. ^f^ *WPI* Widespread Pain Index. ^g^ *SSS* Symptoms Severity Scale. ^h^ *PSD* Polysymptomatic Distress Scale

## Discussion

The main finding of this study is that the prevalence of PC in FM patients appears to be similar to the one found in RD patients, which suggests that FM patients do not have an increased risk of developing PC. The frequency of PC and the clinical picture in the total population of our patients (74%) are similar to the ones previously described for the general population (between 40 and 80%) [3, 4].

The possible pathogenesis of PC is not known, but some theories have been proposed to explain the persistence of the symptoms. These include: residual damage consequences of the severe inflammatory process during the acute phase of the disease [17], persistence of the virus in some structures of the organism [18], or an autoimmune phenomenon triggered by de SAR-COV-2 infection [19]. These three mechanisms may coexist in PC pathogenesis.

Sequelae secondary to COVID-19 are more frequent in the most severe cases of the disease where the percentage of patients with structural organ damage are also clearly higher [20]. Sequelae are lower [21] in those patients with a more benign form of the disease but some of these sequelae can also be observed in asymptomatic patients [22].

In our study, the RD group had a more severe COVID-19 with a higher number of x-ray pneumonias and hospital admissions, but the prevalence of PC was similar to the one in the FM group of patients. Other studies have not found any correlation between the severity of COVID-19 and the development of PC, and patients admitted to the hospital for a severe COVID-19 treatment had the same probability of developing PC compared to patients with a mild form of the disease [23].

The persistence of the virus in the organism or the autoimmune phenomenon triggered by the SAR-COV-2 infection induces neuroinflammation with an increase in the permeability of the brain blood barrier and the intracerebral increase of some pro inflammatory peptides and cytokines that may be responsible for the symptoms of PC [24]. Some evidence exists about a similar process of neuroinflammation in FM [25] or EM/CFS [26], and the most common symptoms of these diseases -such as fatigue, pain, cognitive impairment, sleep alterations or headache- are also present in our patients, with PC suggesting a similar pathogenetic mechanism of neuroinflammation.

At present, the duration of PC is not known but it is plausible that the symptoms improve with time [4, 20]. We found that the RD patients had a small but significant longer duration of the disease (3 months difference) than the FM patients, but we believe that this aspect is not relevant for our study because the prevalence of PC was similar in both groups. Some studies have shown that patients may be still symptomatic after 1 year and no clear predictive variables are associated with this long persistence of the symptoms [20, 27].

In our FM group of patients the number of symptoms during the acute infection, the impairment after COVID-19, the number of PC symptoms and the severity of fatigue, waking unrefreshed and cognitive impairment were significantly higher than in the RD group. It is well known that the comorbidity with FM may increase the severity of other comorbid diseases. For example, in rheumatoid arthritis some of the principal outcome measures are worse when the patient also has FM, although objective measures of severity such as erythrocyte sedimentation rate, C reactive protein or articular erosions do not show any differences with the patients without FM [28]. In other inflammatory rheumatic diseases, such as ankylosing spondylitis [29], similar findings have been described when there is also comorbidity with FM. PC symptoms were almost identical in both groups of patients, although more frequent and severe in the FM group.

A previous study [30] has shown that global FM symptoms are more severe in patients with COVID-19. However, in the FM patients with PC in our study, we did not find any difference in PSD scores before and after COVID-19, which suggests that PC did not impair the severity of FM. We also measured the global impact of FM on patients by means of the total ICAF score and its factors [15]. The unique factor that impairs after COVID-19 was the physical factor, which measures physical activity and it is clearly influenced by fatigue. However, emotional and coping factors did not change, which again suggests that there was no aggravation of FM in these patients.

As to the autoimmune pathogenic mechanism, it is known that the SARS-COV-2 infection may produce multiple antibodies against different proteins of the organism and the persistence of these antibodies after the infection may explain PC [31]. In a recent study performed in a murine model of FM [32], it was observed that the infusion of purified immunoglobulins of FM patients in mice triggered symptoms of FM, which suggests the possible autoimmune origin of this disease. According to this hypothesis, FM and PC may share similar pathogenic mechanisms.

To our knowledge, this is the first study that analyses the prevalence and the principal characteristics of PC in FM patients. PC is considered nowadays an important health issue in the general population due to its high prevalence and the severe consequences for the patients’ quality of life. It is known that FM patients have a more symptomatic COVID-19 [30]; therefore, the issue of whether these patients may develop more PC is of special relevance.

The main limitation of this study is that we have not evaluated the presence of comorbid FM in the RD group of patients. Since the global prevalence of FM in the main inflammatory and autoimmune diseases is high [33], the evaluation of the principal variables in this group of patients may be biased. However, subjective variables in RD patients in our study were lower than in the FM group of patients, which suggests that this possible bias did not impact overall study findings.

Another limitation of our work is the retrospective design with all the corresponding limitations of this type of studies.

The relative small number of patients in both groups may also be a limitation of this work. Although there was no statistical difference in prevalence between the FM and the RD groups, a certain trend towards a higher increase of PC in the FM group was observed, which could reach statically significance if the sample size were higher.

## Conclusions

The prevalence of PC is similar in patients with FM in comparison with other inflammatory and autoimmune rheumatic diseases. Although the symptoms of PC are similar to FM, our data suggest that PC and FM are two distinct diseases that may share similar pathogenic mechanisms.

## Data Availability

The datasets used and/or analysed during the current study are available from the corresponding author on reasonable request.

## Abbreviations

ACR: American College of Rheumatology
ANOVA: Analysis of variance
COVID-19: Coronavirus disease 2019
DF: Degree of freedom
FM: Fibromyalgia
HGUGM: Hospital General Universitario Gregorio Marañón
ICAF: Combined Index of Severity in Fibromyalgia
IiSGM: Instituto de Investigación Sanitaria Gregorio Marañón
ME/CFS: Myalgic Encephalomyelitis/Chronic Fatigue Syndrome
N: Number of patients
PC: Post-COVID-19
PCR: Protein chain reaction
PSD: Polysymptomatic Distress Scale
PGIC: Patient Global Impression of Change
RD: Rheumatic diseases
SARS-COV-2: Severe Acute Respiratory Syndrome Coronavirus-2
SD: Standard deviation
SSS: Symptoms Severity Scale
WPI: Widespread Pain Index

## References

[1] Callard F, Perego E (2021). How and why patients made long Covid. Soc Sci Med.

[2] Carfi A, Bernabei R, Landi F (2020). Persistent symptoms in patients after acute COVID-19. JAMA..

[3] Nalbandian A, Sehgal K, Gupta A, Madhavan MV, McGroder C, Stevens JS (2021). Post-acute COVID-19 syndrome. Nat Med.

[4] Whitaker M, Elliott J, Chadeau-Hyam M, Riley S, Darzi A, Cooke G, et al. Persistent COVID-19 symptoms in a community study of 606,434 people in England. Nat Commun. 2022;13(1):1957. 10.1038/s41467-022-29521-z PMC9005552 35413949

[5] Dennis A, Wamil M, Kapur S, Alberts J, Badley AD, Decker GA, et al. Multi-organ impairment in low-risk individuals with long COVID [internet]. Health Policy. 2020; Oct [cited 2021 Aug 21]. Available from: http://medrxiv.org/lookup/doi/10.1101/2020.10.14.20212555.

[6] Klein H, Asseo K, Karni N, Benjamini Y, Nir-Paz R, Muszkat M (2021). Onset, duration and unresolved symptoms, including smell and taste changes, in mild COVID-19 infection: a cohort study in Israeli patients. Clin Microbiol Infect.

[7] Davis HE, Assaf GS, McCorkell L, Wei H, Low RJ, Re’em Y, et al. Characterizing long COVID in an international cohort: 7 months of symptoms and their impact. EClinicalMedicine. 2021;38:101019. 10.1016/j.eclinm.2021.101019 PMC8280690 34308300

[8] Rasa S, Nora-Krukle Z, Henning N, Eliassen E, Shikova E, The European Network on ME/CFS (EUROMENE) (2018). Chronic viral infections in myalgic encephalomyelitis/chronic fatigue syndrome (ME/CFS). J Transl Med.

[9] Baio P, Brucato A, Buskila D, Gershwin ME, Giacomazzi D, Lopez LR (2008). Autoimmune diseases and infections: controversial issues. Clin Exp Rheumatol.

[10] Moldofsky H, Patcai J (2011). Chronic widespread musculoskeletal pain, fatigue, depression and disordered sleep in chronic post-SARS syndrome; a case-controlled study. BMC Neurol.

[11] Hickie I, Davenport T, Wakefield D, Vollmer-Conna U, Cameron B, Vernon SD (2006). Post-infective and chronic fatigue syndromes precipitated by viral and non-viral pathogens: prospective cohort study. BMJ..

[12] Wolfe F, Clauw DJ, Fitzcharles M-A, Goldenberg DL, Katz RS, Mease P (2010). The American College of Rheumatology Preliminary Diagnostic Criteria for fibromyalgia and measurement of symptom severity. Arthritis Care Res.

[13] Rampakakis E, Ste-Marie PA, Sampalis JS, Karellis A, Shir Y, Fitzcharles M-A (2015). Real-life assessment of the validity of patient global impression of change in fibromyalgia. RMD Open.

[14] Vallejo MA, Rivera J, Esteve-Vives J, Icaf G (2010). Development of a self-reporting tool to obtain a combined index of severity of fibromyalgia (ICAF*). Health Qual Life Outcomes.

[15] Vallejo MA, Rivera J, Esteve-Vives J, Rejas J, Group ICAF (2011). A confirmatory study of the combined index of severity of fibromyalgia (ICAF*): factorial structure, reliability and sensitivity to change. Health Qual Life Outcomes.

[16] Wolfe F, Clauw DJ, Fitzcharles M-A, Goldenberg DL, Häuser W, Katz RS (2011). Fibromyalgia criteria and severity scales for clinical and epidemiological studies: a modification of the ACR preliminary diagnostic criteria for fibromyalgia. J Rheumatol.

[17] Hadjadj J, Yatim N, Barnabei L, Corneau A, Boussier J, Smith N (2020). Impaired type I interferon activity and inflammatory responses in severe COVID-19 patients. Science..

[18] Dias De Melo G, Lazarini F, Levallois S, Hautefort C, Michel V, Larrous F, et al. COVID-19-associated olfactory dysfunction reveals SARS-CoV-2 neuroinvasion and persistence in the olfactory system [internet]. Neuroscience. 2020 Nov [cited 2021 Aug 26]. Available from. 10.1101/2020.11.18.388819.

[19] Ramos-Casals M, Brito-Zerón P, Mariette X. Systemic and organ-specific immune-related manifestations of COVID-19. Nat Rev Rheumatol [Internet]. 2021; Apr 26 [cited 2021 May 4]; Available from: http://www.nature.com/articles/s41584-021-00608-z. 10.1038/s41584-021-00608-z PMC8072739 33903743

[20] Huang L, Yao Q, Gu X, Wang Q, Ren L, Wang Y (2021). 1-year outcomes in hospital survivors with COVID-19: a longitudinal cohort study. Lancet.

[21] Daugherty SE, Guo Y, Heath K, Dasmariñas MC, Jubilo KG, Samranvedhya J (2021). Risk of clinical sequelae after the acute phase of SARS-CoV-2 infection: retrospective cohort study. BMJ..

[22] Eiros R, Barreiro-Perez M, Martin-Garcia A, Almeida J, Villacorta E, Perez-Pons A, et al. Pericarditis and myocarditis long after SARS-CoV-2 infection: a cross-sectional descriptive study in health-care workers [internet]. Cardiovascular Medicine. 2020 Jul [cited 2021 Aug 29]. Available from:. 10.1101/2020.07.12.20151316.

[23] Blomberg B, Mohn KG-I, Brokstad KA, Zhou F, Linchausen DW, Hansen B-A, et al. Long COVID in a prospective cohort of home-isolated patients. Nat Med [Internet]. 2021; Jun 23 [cited 2021 Jun 30]; Available from: http://www.nature.com/articles/s41591-021-01433-3. 10.1038/s41591-021-01433-3 PMC8440190 34163090

[24] Rhea EM, Logsdon AF, Hansen KM, Williams LM, Reed MJ, Baumann KK (2021). The S1 protein of SARS-CoV-2 crosses the blood–brain barrier in mice. Nat Neurosci.

[25] Littlejohn G, Guymer E (2018). Neurogenic inflammation in fibromyalgia. Semin Immunopathol.

[26] VanElzakker MB, Brumfield SA, Lara Mejia PS (2019). Neuroinflammation and cytokines in Myalgic encephalomyelitis/chronic fatigue syndrome (ME/CFS): a critical review of research methods. Front Neurol.

[27] Seeßle J, Waterboer T, Hippchen T, Simon J, Kirchner M, Lim A (2021). Persistent symptoms in adult patients one year after COVID-19: a prospective cohort study. Clin Infect Dis.

[28] Coury F, Rossat A, Tebib A, Letroublon M-C, Gagnard A, Fantino B (2009). Rheumatoid arthritis and fibromyalgia: a frequent unrelated association complicating disease management. J Rheumatol.

[29] Aloush V, Ablin JN, Reitblat T, Caspi D, Elkayam O (2007). Fibromyalgia in women with ankylosing spondylitis. Rheumatol Int.

[30] Salaffi F, Giorgi V, Sirotti S, Bongiovanni S, Farah S, Bazzichi L, et al. The effect of novel coronavirus disease-2019 (COVID-19) on fibromyalgia syndrome. Clin Exp Rheumatol. 2021;39 Suppl 130(3):72–7. 10.55563/clinexprheumatol/dnxtch 33200740

[31] Chang SE, Feng A, Meng W, Apostolidis SA, Mack E, Artandi M, et al. New-onset IgG autoantibodies in hospitalized patients with COVID-19 [internet]. Allerg Immunol. 2021 Jan [cited 2021 Aug 28]. Available from:. 10.1101/2021.01.27.21250559. 10.1038/s41467-021-25509-3 PMC8440763 34521836

[32] Goebel A, Krock E, Gentry C, Israel MR, Jurczak A, Urbina CM (2021). Passive transfer of fibromyalgia symptoms from patients to mice. J Clin Investig.

[33] Haliloglu S, Carlioglu A, Akdeniz D, Karaaslan Y, Kosar A (2014). Fibromyalgia in patients with other rheumatic diseases: prevalence and relationship with disease activity. Rheumatol Int.

